# First contact of care for persons with spinal cord injury: a general practitioner or a spinal cord injury specialist?

**DOI:** 10.1186/s12875-021-01547-0

**Published:** 2021-10-02

**Authors:** Dima Touhami, Mirjam Brach, Stefan Essig, Elias Ronca, Isabelle Debecker, Inge Eriks-Hoogland, Anke Scheel-Sailer, Nadja Münzel, Armin Gemperli

**Affiliations:** 1 Department of Health Sciences and Medicine, University of Lucerne, 6002 Lucerne, Switzerland; 2 Swiss Paraplegic Research, 6207 Nottwil, Switzerland; 3 Center of Primary and Community Care, University of Lucerne, 6002 Lucerne, Switzerland; 4 REHAB Basel, 4055 Basel, Switzerland; 5 Swiss Paraplegic Center, 6207 Nottwil, Switzerland; 6 ParaHelp, 6207 Nottwil, Switzerland

**Keywords:** General practitioner, SCI-specialist, Spinal cord injury, First contact of care, Secondary health conditions

## Abstract

**Background:**

Although general practitioners (GPs) are generally considered as the first point of contact for care, this may be different for persons with complex conditions, such as those with spinal cord injury (SCI). The objective of this study is to understand the differences in long-term care provision by GPs and SCI-specialists, by examining (1) the first contact of care for SCI health problems, (2) the morbidity profile and use of health-care services in relation to first contact, and (3) the factors associated with the choice of first contact.

**Methods:**

In this cross-sectional study based on data derived from the Swiss Spinal Cord Injury Cohort Study Community Survey 2017, the main outcome measure was the reported first contact for SCI-specific care. This information was analysed using the chi-square test and logistic regression analysis of groups based on patient characteristics, use of health-care services and secondary health conditions assessed using the Spinal Cord Injury Secondary Conditions Scale (SCI-SCS).

**Results:**

Out of 1294 respondents, 1095 reported their first contact for SCI-specific care; 56% indicated SCI-specialists and 44% specified GPs. On average, participants who first contacted a GP reported higher number of GP consultations (5.1 ± 5.2 vs. 3.9 ± 7.2), planned visits to ambulatory clinics (3.7 ± 7.3 vs. 3.6 ± 6.7) and hospital admissions (GP, 1.9 ± 1.7 vs. 1.5 ± 1.3), but lower number of visits to SCI-specialists (1.7 ± 1.8 vs. 2.6 ± 1.7) and of hospital days (22.8 ± 43.2 vs. 31.0 ± 42.8). The likelihood to contact a GP first was significantly higher in persons ≥75 years old (OR = 4.44, 95% CI = 1.85–10.69), Italian speakers (OR = 5.06, 95% CI = 2.44–10.47), had incomplete lesions (OR = 2.39, 95% CI = 1.71–3.35), experiencing pain (OR = 1.47, 95% CI = 1.04–2.09) or diabetes mellitus (OR = 1.85, 95% CI = 1.05–3.27), but lower for those situated closer to SCI centres (OR = 0.69, 95% CI = 0.51–0.93) or had higher SCI-SCS scores (OR = 0.92, 95% CI = 0.86–0.99).

**Conclusion:**

Age, language region, travel distance to SCI centres, lesion completeness, and occurrence of secondary conditions play a significant role in determining the choice of first contact of care, however there is still some unwarranted variation that remains unclear and requires further research.

## Background

Chronic spinal cord injury (SCI) is a complex medical condition [1], as affected persons experience limitations in their sensory, motor, and autonomic functions that put them at high risk of developing life-threatening complications [2]. Hence, persons with SCI require a first contact of care who can address their acute and chronic medical problems, as well as lifestyle problems [3, 4]. Primary care is recognized as the first point of entry into the health-care system [5]. Accordingly, the choice of which practitioner to contact initially relies on how primary care is defined [6]. According to the European definition of general practice/family medicine, general practitioners (GPs) are the first point of contact for ‘all health problems regardless of the age, sex, or any other characteristic of the person concerned’ [7]. However, persons with SCI may also choose to consult a practitioner other than a GP, such as an SCI-specialist, for diverse health-care needs, and the specialist may then be the one managing and coordinating all aspects of care throughout their lives [4].

Persons with SCI find various ways to receive care for their complex medical needs [4]. A study of the primary care of persons with SCI in the United States, Canada and Britain showed that GPs were more likely to be visited for the treatment of general health problems or for screening [8], and SCI-specialists were mainly consulted for routine annual follow-ups or the treatment of SCI-specific conditions, such as bowel and bladder problems, urinary tract infections, and pressure injuries [8]. In Switzerland, GPs were consulted more often for acute problems, and SCI-specialists were contacted for follow-up care [9]. However, specialist-focused health-care provision is problematic when the geographical distribution of physicians is not even across regions [10]. That is, SCI-specialists are likely to be situated in urban areas or in close proximity to hospitals and specialized centres. This may lead to geographically variable, poorly distributed access to care and unmet health-care needs—a problem that is particular to those living in rural areas or those with limited access to long-distance transportation [11]. Further, relying mostly on GPs in the absence of regional specialized care may lead to disparities in caring for the complex conditions of persons with SCI [12].

The fragmentation of services and the lack of integrated care across physicians also pose a challenge to meeting the health-care needs of persons with complex medical conditions [13]. Therefore, greater medical support and the concept of ‘shared care’ are needed to achieve positive health outcomes [14–16]. Efforts have been made to reach a consensus on health concepts and modes of providing primary care for persons with SCI, but these have not been successful [4]. Nonetheless, some health organizations have succeeded in constructing models integrating primary and specialty care to improve access, care coordination and health outcomes for the SCI population [4, 17].

In Switzerland, a shift towards inter-professional collaboration between GPs and SCI-specialists is being realized [18] with the aim of providing integrated long-term care for persons with SCI living in the community. The present study focuses on this model, with the aim of understanding the differences in long-term care provision by GPs and SCI-specialists. To this end, it examines (1) the first contact of care for SCI-related health problems, (2) the morbidity profile and use of health-care services in relation to the first contact, and (3) the factors associated with the choice of first contact.

## Methods

### Study design

Cross-sectional data were derived from the national community survey of the Swiss Spinal Cord Injury Cohort Study (SwiSCI) conducted between March 2017 and March 2018. Persons with SCI aged over 16 years and living in Switzerland (*N* = 3959) were invited to participate. Details on the methodology and study population of the Swiss national community survey of functioning after SCI are described by Gross-Hemmi et al. [19].

### Measures

#### Outcome variables

First contact of care was determined based on the survey question ‘Who is your first point of contact for health problems related to your spinal cord injury?’ The choices provided were GP, SCI-specialist, and other. A binary variable was then constructed with GPs comprising one group and SCI-specialists comprising the other group.

#### Explanatory variables

1. Participants’ characteristics

The sociodemographic characteristics of the participants used in the study were sex, age groups according to the International Spinal Cord Society recommendations [20], civil status, availability of informal caregivers, place of birth, years of formal education and employment status [21]. Subjective social status was defined using the MacArthur scale and constructed as a categorical variable based on the level of social status: low (rungs 1–3), middle (rungs 4–7) and high (rungs 8–10) [22]. The geographical information recorded included the national language of responses to the questionnaire (German, French or Italian) and distance travelled by vehicle to personal GP and nearest SCI centre. Time to the nearest SCI centre was calculated using the Google Maps Directions API and constructed as a binary variable [23]. The SCI characteristics included were time since injury in years, level of injury in patients with tetraplegia (cervical spine level) or paraplegia (damage at the level of the thorax or below), and lesion severity (complete or incomplete loss of sensory and/or motor functions below the level of injury). Cause of SCI was categorized as traumatic (insult caused by an external force) or non-traumatic (injury caused by an underlying pathology) [20].

(2) Morbidity profile

The morbidity profile of the study participants was examined using the Spinal Cord Injury Secondary Conditions Scale (SCI-SCS) [24]. Participants were asked to report their health problems over the previous 3 months on a scale of 0 (non-existent or mild) to 3 (major or chronic). The morbidity profile represented secondary health conditions, which were self-rated as a moderate or major concern, and other existing conditions, such as coronary heart disease, cancer, depression and sleep problems. SCI-SCS total score ranged from 0 to 48, and it was based on the sum of the problem ratings. Higher scores denoted a greater number of problems with secondary conditions [24].

(3) Use of health-care services

Participants were asked to indicate if during the 12 months prior to the survey they had visited: (1) any of eight providers (GP, SCI-specialist, other specialist, nurse or midwife, psychologist, speech therapist, occupational therapist or physiotherapist) and the frequency of visits; (2) ambulatory clinics (for planned or unplanned visits) and the frequency of visits; (3) SCI centres (for control or ambulatory treatment); and (4) in-patient facilities (such as a hospital or an SCI centre), the frequency of visits, and the total number of days in hospital.

(4) Factors associated with the choice of first contact

Sociodemographic and geographic features, SCI characteristics, secondary conditions, and other chronic conditions were analysed as predictors of the first contact of care.

### Statistical methods

Descriptive statistical data about participants’ characteristics, their morbidity profile and use of health-care services were reported as absolute and relative frequencies, means and standard deviations, medians, and 1st and 3rd quartiles. To measure the association of first contact for SCI-specific care and use of services, the *t*-test and Pearson’s chi-square test were used respectively. Univariate and multivariable logistic regression analyses were conducted to identify factors related to first contact of care. Multiple imputation (MI), assuming missing at random, was used to account for potential biases caused by item non-response and missing values. Odds ratios (ORs) and 95% confidence intervals (CIs) were reported. All statistical findings were compared at a significance level of *P* < 0.05, and all statistical analyses were performed using Stata® version 16.0 (Stata Corp LP, College Station, Texas, USA).

## Results

Out of the 3959 patients who were invited, 1294 (response rate, 33%) participated in the SwiSCI community survey. Of those, 1095 (85%) reported a first contact for SCI-specific care and were included in the analysis.

### Participants’ characteristics

The baseline characteristics of the study population are shown in Table 1. Of the 1095 participants, 72% were male, and the mean age was 55.8 years (±14.5). German was the language of response for 70% of the participants. With regard to the geographical data, 86 and 31% of the participants were living within 25 min of vehicle-driving distance of their personal GP (12 ± 11.7 min) and the nearest SCI centre (38 ± 21.1 min) respectively. Overall, 30% of the participants reported having tetraplegia, 33% reported complete lesions, and 80% reported trauma-induced SCI.

**Table 1:** Characteristics of the study population

Variable	Value (*N* = 1095)
Males, n (%)	787 (72)
Age groups, n (%)	
*16–29 years*	48 (4)
*30–44 years*	204 (19)
*45–59 years*	376 (34)
*60–74 years*	358 (33)
*≥75 years*	109 (10)
Married^a^, n (%)	590 (54)
Informal caregiver^b^, n (%)	498 (46)
Born in Switzerland, n (%)	909 (83)
Years of education, n (%)	
*Less than 10 years*	65 (6)
*10–12 years*	206 (20)
*13–15 years*	438 (42)
*≥16 years*	342 (32)
Employed, n (%)	510 (47)
Subjective social status^c^, n (%)	
*Low (rungs 1–3)*	144 (14)
*Middle (rungs 4–7)*	738 (70)
*High (rungs 8–10)*	166 (16)
Language, n (%)	
*German*	769 (70)
*French*	279 (26)
*Italian*	47 (4)
Travel distance to health-care provider	
*≤25 min travel distance to personal GP*^d^*, n (%)*	913 (86)
*≤25 min travel distance to nearest SCI centre*^d^*, n (%)*	334 (31)
Time since SCI in years, median (Q1–Q3)	15.7 (7.8–27.2)
Tetraplegia, n (%)	324 (30)
Complete injury, n (%)	361 (33)
*Missing*	120 (11)
Traumatic SCI, n (%)	872 (80)

*SCI* Spinal cord injury, *Q1–Q3* 1st to 3rd quartile

The number of missing observations is less than 5% for all characteristics, unless indicated otherwise

^a^Married denotes those reporting being married or in a registered partnership

^b^ Informal caregiver is a family member or friend who provides unpaid care

^c^ SSS is the subjective social status based on MacArthur Scale classification

^d^Travel distance is the distance by motor vehicle

### Morbidity profile

Table 2 shows the self-reported health problems persons with SCI had experienced within the previous 3 months. Overall, the mean SCI-SCS score was 14 (±7.5). For SCI-specific care, 56% (*n* = 612) of the participants reported that their first contact was an SCI-specialist and 44% (*n* = 483) stated their first contact was a GP.

**Table 2:** Morbidity profile of the study participants according to the first contact of care

		First contact of care		
		GP (*n* = 483)	Specialist (*n* = 612)	
	*N* = 1095	Moderate/ Major problem^1^	Moderate/ Major problem^1^	Statistical significance^2^
SCI-SCS score, (mean ± SD)	(14.0 ± 7.5)	(13.1 ± 7.7)	(14.6 ± 7.3)	††
Genitourinary and bowel				
*Bowel dysfunction, n (%)*	469 (43)	187 (40)	282 (60)	*
*Bladder dysfunction, n (%)*	488 (45)	200 (41)	288 (59)	
*Sexual dysfunction, n (%)*	608 (56)	242 (40)	366 (60)	**
*Urinary tract infections, n (%)*	389 (36)	155 (40)	234 (60)	*
Muscle structure and pain				
*Contractures, n (%)*	378 (35)	162 (43)	216 (57)	
*Pain, n (%)*	620 (57)	274 (44)	346 (56)	
*Muscle spasm (spasticity), n (%)*	518 (47)	204 (39)	314 (61)	**
Skin, breathing and metabolism				
*Pressure injuries, n (%)*	176 (16)	73 (41)	103 (59)	
*Diabetes mellitus, n (%)*	71 (6)	45 (63)	26 (37)	**
*Respiratory problems, n (%)*	111 (10)	49 (44)	62 (56)	
*Injury due to loss of sensation, n (%)*	100 (9)	39 (39)	61 (61)	
Circulatory and autonomic problems				
*Autonomic dysreflexia, n (%)*	145 (13)	52 (36)	93 (64)	*
*Postural hypotension, n (%)*	87 (8)	32 (37)	55 (63)	
*Circulatory problems, n (%)*	219 (20)	92 (42)	127 (58)	
*Heterotopic bone ossification, n (%)*	42 (4)	22 (52)	20 (48)	
Other chronic conditions				
*Coronary heart disease, n (%)*	112 (10)	62 (55)	50 (45)	*
*Cancer, n (%)*	73 (7)	37 (51)	36 (49)	
*Depression, n (%)*	154 (14)	72 (47)	82 (53)	
*Sleep problems, n (%)*	384 (35)	158 (41)	226 (59)	

*GP* General practitioner, *SCI* Spinal Cord Injury; Specialist represents SCI-specialist with own office or specialist of one of the SCI centres

SCI-SCS score: Spinal cord injury secondary conditions scale total score (0 to 48). Higher scores reflect a greater number of problems with secondary conditions

^1^ Represents [the percentage of] participants with moderate or major health condition who choose GP/specialist as first contact of care. 100% is the overall number of participants with the corresponding moderate or major health condition

^2^ Pearson’s Chi^2^ test is used to test significance between first contact of care and major health conditions. * *P*-value < 0.05, ** *P-*value < 0.01, *** *P*-value < 0.001. T-test is used for testing difference of SCI-SCS score means between the two groups. ^†^ *P*-value < 0.05, ^††^ *P*-value < 0.01, ^†††^ *P*-value < 0.001

The SCI-SCS mean score for persons who initially consulted a GP (13.1 ± 7.7) was significantly lower than that in the SCI-specialist group (14.6 ± 7.3). The majority of SCI persons with autonomic dysreflexia (64%), muscle spasm (61%), sexual dysfunction (60%), urinary tract infections (60%) and bowel dysfunction (60%) contacted an SCI-specialist first for SCI-related health problems. With regard to other health conditions, more than half of the participants with diabetes mellitus (63%) and coronary heart disease (55%) consulted a GP first for SCI-specific care.

### Use of health-care services

Table 3 shows the use of health-care services within the previous 12 months based on first contact of care. Most of the participants (87%) who visited health-care professionals, consulted a GP in this period (mean 4.5, SD ± 6.4). Those who consulted an SCI-specialist first were more likely to visit SCI-specialists (78%), occupational therapists (70%), physiotherapists (60%), ambulatory clinics for unplanned visits (59%), GPs (53%) and SCI centres for ambulatory treatment (80%), control visits (75%) or for in-patient stays (77%).

**Table 3:** Use of health-care services in total and according to the first contact of care

Utilization of health-care services in the previous year		First contact of care						
		GP			Specialist^*1*^			
		Visits^*2*^	Number of visits	Number of days	Visits^*2*^	Number of visits	Number of days	Statistical significance^*3*^
	n (%)	n (%)	mean (SD)	mean (SD)	n (%)	mean (SD) ^4^	Mean (SD)	
**Total**, n (%)	**1095 (100)**	**483 (44)**			**612 (56)**			
**Health-care professionals**	**1051 (96)**	**456 (43)**			**595 (57)**			
*General practitioner*	909 (87)	430 (47)	5.1 (5.2)		479 (53)	3.9 (7.2)		^††^ ***
*Specialist for paraplegia*	463 (44)	101 (22)	1.7 (1.8)		362 (78)	2.6 (3.7)		^†^ ***
*Other specialist*	561 (53)	235 (42)	5.3 (9.8)		326 (58)	4.5 (7.2)		
*Nurse or midwife*	60 (6)	30 (50)	105.1 (156.0)		30 (50)	40.7 (86.7)		
*Psychologist*	97 (9)	44 (45)	10.5 (12.2)		53 (55)	11.9 (14.3)		
*Speech therapist*	15 (1)	6 (40)	19.8 (17.3)		9 (60)	31.3 (43.9)		
*Occupational therapist*	176 (17)	52 (30)	18.6 (25.1)		124 (70)	21.3 (29.7)		***
*Physiotherapist*	685 (65)	273 (40)	44.1 (36.6)		412 (60)	47.9 (36.0)		***
**Ambulatory or polyclinic visits**	**567 (53)**	**205 (36)**			**362 (64)**			
*Planned*	446 (79)	151 (34)	3.7 (7.3)		295 (66)	3.6 (6.7)		***
*Unplanned*	204 (36)	83 (41)	1.6 (1.1)		121 (59)	1.9 (3.7)		
**SCI centres**	**639 (59)**	**169 (26)**			**470 (74)**			
*Control visit*	570 (89)	141 (25)			429 (75)			***
*Ambulatory treatment*	197 (31)	40 (20)			157 (80)			***
**Inpatient stay**	**344 (31)**	**133 (39)**			**211 (61)**			
*Hospital*	331 (96)	129 (39)	1.9 (1.7)	22.8 (43.2)	202 (61)	1.5 (1.3)	31.0 (42.8)	*
*SCI centre*	151 (44)	35 (23)			116 (77)			***
**No visits to any health service provider**	**35 (3)**	**21(60)**			**14 (40)**			

*GP* general practitioner, *SCI* spinal cord injury, *SD* standard deviation

^1^ SCI-specialist with their own office or a specialist at one of the SCI centres

^2^ 100% represents the overall number of participants with the corresponding visits to health-care services

^3^ Pearson’s chi-square test was used to analyse the significance of differences in the choice of first contact of care and visits to health-care services. *P-value < 0.05, **P-value < 0.01, ***P-value < 0.001. T-test is used for testing the difference of means between the two groups. ^†^*P-*value < 0.05, ^††^*P-*value < 0.01, ^†††^*P-*value < 0.001

Compared to those who initiated contact with an SCI-specialist, participants who first contacted a GP reported a higher number of GP consultations (GP, 5.1 ± 5.2; specialist, 3.9 ± 7.2), planned visits to ambulatory clinics (GP, 3.7 ± 7.3; specialist, 3.6 ± 6.7) and hospital admissions (GP, 1.9 ± 1.7; specialist, 1.5 ± 1.3), but lower number of visits to SCI-specialists (GP, 1.7 ± 1.8; specialist, 2.6 ± 1.7) and of hospital days (GP, 22.8 ± 43.2; specialist, 31.0 ± 42.8). Using t-test, statistically significant differences were only found in the number of visits to GPs (*P* < 0.01) and SCI-specialists (*P* < 0.05).

### Factors associated with choice of GP as the first contact for SCI-specific care

Multivariable logistic regression (Table 4) showed that the likelihood of the first contact being a GP rather than an SCI-specialist for SCI-specific care was significantly higher in those who were ≥ 75 years old (OR = 4.44, 95% CI = 1.85–10.69) or Italian speaking (OR = 5.06, 95% CI = 2.44–10.47), as compared with persons below 29 years of age and German-speaking persons. SCI persons with incomplete lesions (OR = 2.39, 95% CI = 1.71–3.35), pain (OR = 1.47, 95% CI = 1.04-2.09) or a history of diabetes mellitus (OR = 1.85, 95% CI = 1.05–3.27) were significantly more likely to first contact a GP than persons with complete lesions, not experiencing pain or with no diabetes respectively. Those residing in close proximity to SCI centres (OR = 0.69, 95% CI = 0.51–0.93) or who had higher SCI-SCS total score (OR = 0.92, 95% CI = 0.86–0.99) were significantly less likely to initially consult a GP for SCI-related health problems.

**Table 4:** Factors associated with choice of GP as the first contact for SCI-specific care

Factors	Univariate OR (95% CI)	Multivariable OR (95% CI)
Sociodemographic factors		
Males, *(ref: females)*	0.86 (0.66–1.12)	0.97 (0.70–1.35)
Age groups, *(ref:* 16–29 *years)*		
*30–44 years*	0.99 (0.50–1.95)	1.08 (0.52–2.23)
*45–59 years*	1.50 (0.79–2.85)	1.41 (0.69–2.89)
*60–74 years*	2.14 (1.12–4.08) *	1.62 (0.76–3.47)
*≥ 75 years*	5.11 (2.47–10.61) ***	4.44 (1.85–10.69) **
Married^a^, *(ref: single)*	1.29 (1.01–1.64) *	1.07 (0.79–1.45)
Informal caregiver, *(ref: paid assistance or none)*^b^	0.84 (0.66–1.07)	0.80 (0.59–1.09)
Born in Switzerland, *(ref: foreign born)*	1.02 (0.73–1.41)	1.22 (0.83–1.80)
Years of education, *(ref: < 10 years)*		
*10–12 years*	0.80 (0.46–1.39)	0.94 (0.50–1.76)
*13–15 years*	0.68 (0.40–1.16)	0.85 (0.47–1.54)
*≥ 16 years*	0.44 (0.25–0.77) **	0.70 (0.38–1.31)
Employed, *(ref: unemployed)*	0.59 (0.46–0.75) ***	0.90 (0.64–1.26)
Subjective social status, *(ref: low SSS [1–3])*^c^		
*Middle SSS (4–7)*	0.64 (0.44–0.91) *	0.73 (0.47–1.13)
*High SSS (8–10)*	0.65 (0.41–1.02)	0.81 (0.46–1.43)
Income in Swiss Francs^d^, *(ref: less than 2000)*		
*Between 2000 and 2999*	1.20 (0.64–2.23)	1.12 (0.55–2.27)
*Between 3000 and 3999*	0.92 (0.50–1.69)	0.93 (0.47–1.83)
*Between 4000 and 4999*	0.73 (0.40–1.35)	0.81 (0.40–1.63)
*Between 5000 and 6000*	0.62 (0.33–1.14)	0.62 (0.31–1.26)
*More than 6000*	0.61 (0.34–1.11)	0.70 (0.34–1.41)
Geographical factors		
Language, *(ref: German)*		
*French*	1.10 (0.83–1.45)	0.99 (0.72–1.37)
*Italian*	2.44 (1.35–4.43) **	5.06 (2.44–10.47) ***
Travel distance^e^ to personal GP, *(ref: > 25 min)*^g^		
*≤25 min to personal GP*	1.14 (0.81–1.61)	1.22 (0.82–1.81)
Travel distance^e^ to SCI centre, *(ref: > 25 min)*		
*≤ 25 min to nearest SCI centre*	0.88 (0.67–1.14)	0.69 (0.51–0.93) *
Injury-related factors		
Time since injury	0.99 (0.99–1.00)	1.01 (0.99–1.02)
Lesion status, *(ref: paraplegia)*		
*Tetraplegia*	0.95 (0.73–1.22)	0.94 (0.68–1.29)
Severity of injury, *(ref: complete)*		
*Incomplete*	2.60 (1.99–3.37) ***	2.39 (1.71–3.35) ***
Cause of SCI, *(ref: non-traumatic)*		
*Traumatic*	0.55 (0.41–0.73) ***	0.75 (0.52–1.07)
Secondary health conditions factors ^h^		
SCI-SCS score	0.97 (0.95-0.98) ***	0.92 (0.86-0.99) *
Genitourinary and bowel		
*Bowel dysfunction*	0.73 (0.58–0.93) *	0.86 (0.60–1.23)
*Bladder dysfunction*	0.80 (0.63–1.02)	1.21 (0.84–1.76)
*Sexual dysfunction*	0.69 (0.55–0.88) **	1.09 (0.74–1.59)
*Urinary tract infections*	0.76 (0.59–0.98) *	1.10 (0.77–1.56)
Muscle structure and pain		
*Contractures*	0.91 (0.71–1.16)	1.05 (0.73–1.51)
*Pain*	1.00 (0.79–1.26)	1.47 (1.04–2.09) *
*Muscle spasm (spasticity)*	0.71 (0.55–0.90) **	0.95 (0.68–1.34)
Skin, breathing and metabolism		
*Pressure injuries*	0.87 (0.63–1.20)	1.10 (0.71–1.70)
*Diabetes mellitus*	2.22 (1.35–3.64) **	1.85 (1.05–3.27) *
*Respiratory problems*	0.97 (0.64–1.47)	1.14 (0.68–1.90)
*Injury due to loss of sensation*	0.79 (0.52–1.20)	1.23 (0.72–2.07)
Circulatory/autonomic problems		
*Autonomic dysreflexia*	0.68 (0.47–0.97) *	1.08 (0.65–1.79)
*Postural hypotension*	0.71 (0.45–1.11)	0.88 (0.48–1.60)
*Circulatory problems*	0.89 (0.65–1.22)	1.11 (0.73–1.68)
*Heterotopic bone ossification*	1.33 (0.72–2.45)	1.62 (0.77–3.42)
Other chronic conditions		
*Coronary heart disease*	1.59 (1.07–2.35) *	1.22 (0.76–1.93)
*Cancer*	1.30 (0.80–2.11)	0.95 (0.56–1.64)
*Depression*	1.11 (0.80–1.55)	1.33 (0.89–1.98)
*Sleep problems*	0.83 (0.64–1.06)	1.00 (0.70–1.41)

OR: Odd ratios of logistic regression, *P-value < 0.05, **P-value < 0.01, ***P-value < 0.001

*CI* confidence interval, *SCI* spinal cord injury, *GP* general practitioner, *ref*: reference

SCI-SCS score: Spinal cord injury secondary conditions scale total score (0 to 48). Higher scores reflect a greater number of secondary conditions

^a^ Married includes persons in a registered partnership

^b^ Informal caregiver is a family member or friend who provides unpaid care

^c^ SSS is the subjective social status based on MacArthur Scale classification

^d^ Represents the household’s total net income from all sources divided by its equivalent size

^e^ Represents motor vehicle driving distance

^g^ Represents those who are at > 25 min motor vehicle driving distance from their GP or report having no personal GP

^h^ Reference is non-existent or mild

## Discussion

### Main findings

Our study findings demonstrate that persons with SCI who initially contact an SCI-specialist for SCI-specific care have a greater number of problems with secondary conditions and tend to use health-care services more often than those who first consult a GP. Further, persons with SCI who are 75 years of age and older, Italian speakers, those with incomplete lesions, diabetes or experiencing pain, and those living farther from SCI centres are more likely to contact a GP first than an SCI-specialist for SCI-specific care.

### Interpretation and comparison with existing literature

In line with previous studies, our findings show that persons with a greater number of problems with secondary conditions are more likely to seek SCI-specialists, while GPs are the first contact for those with diabetes or experiencing chronic pain [8, 25, 26]. These findings may be partly explained by the limited experience and knowledge of GPs in the area of SCI-specific care: as persons with SCI previously reported unmet information needs, and they often felt more knowledgeable about the management of SCI-specific conditions than their GPs [3, 27]. An additional explanation may be the specialist’s reluctance to manage complex conditions that are far from their own specialties [28]. Conversely, GPs provide care in the context of a holistic approach, which may be better appreciated by persons with chronic health conditions, such as those with diabetes or chronic pain [8, 29]. Nonetheless, the evidence on the number of participants who consulted a GP confirms the complementary role of GPs in the continuity of long-term care for persons with SCI, irrespective of who they first contact. This finding plausibly suggests a possible overlap in care due to a lack of clarity about who provides what care.

In this study, those who initiated contact with SCI-specialists tended to make greater use of health-care services than those who consulted GPs. Although this finding may simply denote that specialists more frequently suggest additional referrals than GPs, it is plausible that age, gender, comorbidities, severity of injury and the rehabilitation stage of participants necessitate more referrals, thereby contributing to the higher frequency of service use [30–32]. An additional factor may be the greater understanding of SCI-specialists about the indications for long-term therapies, such as speech and occupational therapy, counselling and physiotherapy, and the need for comprehensive care. This premise may further explain the two- to three-fold higher use of specialized services in this population than in persons who initiate contact with a GP. Conversely, the lower use of health services by those initially seeking a GP for SCI-specific care may be explained by the GP’s management of complex conditions and referrals. In Switzerland, GPs were reported to manage 94.3% of health problems presented to their practices, with a specialist referral rate of 9.4% [33].Yet, the lower frequency in the use of health-care services and the higher rate of hospital admissions pose important questions regarding the needs of this group and whether these were met, particularly since access to SCI-specific care and periodic comprehensive health evaluations are necessary to avoid preventable complications [34].

In this study, those who were aged 75 or over were most likely to initially consult a GP for their SCI-related health problems. This finding may be explained by the heightened risk of chronic conditions in older persons with SCI, which often requires frequent visits to GPs. As a result, a personal relationship with their physician is developed, and this may, in turn, govern their choice of first contact and trump their need for a disease-specific care approach [28]. Qualitative research in the general population also revealed that the elderly tend to appreciate the social and emotional behaviour exhibited by GPs during visits, and focus less on how care is provided [35]. They appeared to value the information and communications they received about their medical conditions and treatment, and described the care as individualized and tailored to meet their needs [36].

Consistent with previous findings on the same population, our results show that Italian speakers were five times more likely to initiate contact with a GP for SCI-specific care than German and French speakers [12]. It was previously suggested that SCI persons from Italian-speaking regions favoured care by GPs who spoke the same language over specialized treatment in different language settings [37]. Moreover, the geographical dispersal of services, mainly in rural areas, lack of specialized clinics with SCI expertise, and general culture may also contribute to the choice of first contact among Italian speakers.

Our study shows that the travel distance to SCI centres is a significant predictive factor of first contact of care. This finding requires an important consideration, particularly for persons who live in rural areas or are located geographically farther from SCI centres. Their choice of GP for first contact may be out of convenience, or is rather forced upon them due to transportation issues. This may hinder their access to needed specialty care, an argument that is supported by similar findings in a previous study. As described by Ronca et al., persons living farther from SCI centres tend to prefer proximate but less specialized care over comprehensive services at SCI centres [11, 31]. Introducing small outpatient clinics or outreach services were recommended as possible ways to meet the health-care needs of persons living farther from SCI centres and in minority regions [11].

The present findings indicate that study participants with incomplete lesions were more likely to first seek contact with a GP for SCI-specific care than those with complete lesions. This finding is surprising, as it was previously reported that persons with incomplete lesions expressed dissatisfaction with the quality of primary care services in terms of the fulfilment of SCI-specific care needs [11]. However, differences in the need for SCI-specific care and for a broader range of services between persons with incomplete and complete lesions might explain their choice of first contact [38].

### Weaknesses and strengths

To our knowledge, this is the first study to examine the morbidity profile and the use of health-care services in relation to the first contact for SCI-specific care. Nevertheless, this study has several limitations, including a low response rate and the exclusion of 199 participants from the analysis due to missing values for the outcome measure. In addition, as this is a cross-sectional study, we can only build our assumptions by means of associations: that is, we are not in a position to determine the impact of first contact on the use of health-care services and health outcomes for persons with SCI. The study design simplified the complex question of who was the first contact for SCI-specific health problems in persons with SCI. However, the correct choice of first contact is context-dependent and relies on factors that were not captured in our study, such as health insurance plans and gatekeeping schemes, the level of integration and coordination between GPs and specialists, and remoteness of the place of residence. Finally, as our results are based on self-reported data, recall bias and inaccuracies cannot be dismissed. Despite these limitations, our data are derived from the largest SCI survey in Switzerland, and this has made it possible to include those who may not be properly identified in the Swiss health-care system owing to the lack of a national registry.

### Implications

The findings of this study demonstrate that the choice of first contact, severity of secondary conditions and use of health-care services are interrelated. They also reveal the predominance of GPs in the provision of care to the vast majority of persons with SCI, irrespective of first contact. Nonetheless, the specific reasons for which persons with SCI seek GP care remain unclear. This information would provide better insights into which health problems GPs are consulted for, as well as reflect patients’ views on the competence levels of their GPs. In addition, this study highlights potential future areas of research to examine the impact of rurality and/or geographical location on accessing specialized care. Finally, our results could inform new approaches to primary-care delivery that foster well-defined roles of providers and structured referral pathways for both preventive and follow-up SCI care and take into account the complementary roles of both providers in the long-term care of persons with SCI [11].

## Conclusions

This study confirms the complementary roles of GPs and SCI-specialists in the long-term care of persons with SCI in Switzerland. Age, language region, travel distance to SCI centres, lesion completeness, and occurrence of secondary conditions play a significant role in determining the choice of first contact of care, however there is still some unwarranted variation that remains unclear and requires further research.

## Data Availability

Owing to our commitment to SwiSCI study participants and their privacy, datasets generated during the current study are not made publicly available but can be provided by the SwiSCI Study Center on reasonable request (contact@swisci.ch).

## Abbreviations

SCI: Spinal cord injury
GP: General practitioner
SwiSCI: Swiss Spinal Cord Injury Cohort Study
SCI-SCS: Spinal Cord Injury Secondary Conditions Scale
